# Muscle activity and power output recovery: the roles of rest duration and underlying endurance capacity in the wingate test

**DOI:** 10.3389/fphys.2025.1674597

**Published:** 2025-12-11

**Authors:** Yuxiao Hu, Weijun Liu, Qingqing Duan, Zhigang Gong

**Affiliations:** 1 Key Lab of Aquatic Sports Training Monitoring and Intervention of General Administration of Sport of China, Faculty of Physical Education, Jiangxi Normal University, Nanchang, China; 2 College of Arts and Physical Education, Nanchang Jiaotong Institute, Nanchang, China

**Keywords:** post-activation performance enhancement (PAPE), muscle endurance, recovery duration, surface electromyography, wingate test

## Abstract

**Purpose:**

This study aimed to determine the optimal recovery time for maximizing post-activation performance enhancement in resistance-trained males grouped by local muscle endurance, and evaluate its effects on anaerobic performance and neuromuscular activity.

**Methods:**

Twenty-four healthy men were grouped by repetitions completed at 80% of 1 repetition maximum (1RM) back squat, serving as a functional proxy for muscle endurance characteristics: Low-Muscle-Endurance (LME, n = 8, 20.5 ± 1.2 years), Intermediate-Muscle-Endurance (IME, n = 8, 20.3 ± 1.3 years), and High-Muscle-Endurance (HME, n = 8, 20.1 ± 1.1 years). Participants completed a control session (CON) and three experimental trials involving a back squat conditioning protocol (3 × 3 repetitions at 80% 1RM), followed by a 30-s Wingate Anaerobic Test (WAnT) after 3 (T3), 8 (T8), or 12 (T12) minutes of recovery. Surface electromyography (EMG) signals were recorded from the right rectus femoris (RF), biceps femoris (BF), and gastrocnemius lateralis (GL) during WAnT and analyzed for integrated EMG (IEMG) and mean power frequency (MPF).

**Results:**

A clear hierarchy in baseline peak power (PP) was observed across groups, with LME > IME > HME (all p < 0.05). The LME group also demonstrated a higher fatigue index (FI) than both HME and IME groups (all p < 0.05). Regarding the time course of PAPE, PP for the cohort was significantly higher at T12 than at CON (p = 0.021). RF IEMG at T12 was significantly elevated versus CON (p = 0.024), while BF IEMG increased at T8 (p = 0.011). BF MPF was also higher at T12 compared to CON (p = 0.039). No significant recovery effects were observed for mean power (MP).

**Conclusion:**

Individuals stratified by local muscle endurance exhibited distinct baseline anaerobic capacities and fatigue profiles. The optimal PAPE window occurred 8–12 min post-activation, marked by initial biceps femoris neural drive enhancement, followed by peak power and improved neuromuscular efficiency at 12 min. These findings support this practical stratification method for personalizing recovery strategies, linking PAPE magnitude differences to physiological traits reflected in endurance-based grouping.

## Introduction

Post-activation performance enhancement (PAPE) describes the acute improvement in voluntary muscular performance following a conditioning activity, a phenomenon increasingly applied in strength and conditioning regimens (Krzysztofik et al., 2021). Common conditioning activities include high-intensity efforts such as sprinting (Yetter and Moir, 2008; Dello Iacono et al., 2016; Matusiński et al., 2021) and jumping (Dobbs et al., 2019; Dos Santos Silva et al., 2023), often resulting in performance outputs approaching 80%–90% of maximal capacity. Its manifestation, however, is a net outcome of the competition between two opposing factors: fatigue and post-activation potentiation (PAP) (Sale, 2002). PAP is a short-lived (<2 min) increase in muscle contractility, mediated by enhanced myofilament calcium sensitivity and myosin regulatory light chain phosphorylation in type II fibers, leading to an increased rate of force development (Blazevich and Babault, 2019). In contrast, PAPE is a more complex and longer-lasting phenomenon (peaking several minutes post-activity) that is thought to involve a combination of factors, including increased muscle temperature, intramuscular fluid shifts, and potential contributions from spinal-level excitability and motivational changes (Blazevich and Babault, 2019).

The transition from a fatigued state to a potentiated one is critically governed by recovery duration (Boullosa et al., 2020). While an optimal window of 3–12 min is often reported, significant inter-individual variability exists (Hamada et al., 2000; Wilson et al., 2013). A compelling yet underexplored source of this variability is an athlete’s inherent local muscle endurance capacity. This capacity, functionally assessed via repetitions to failure at 80% 1RM, reflects fatigue resistance, a trait fundamentally linked to muscle fiber typology. We propose that this performance metric offers a more direct and practical predictor of an individual’s recovery kinetics than estimations of fiber-type composition alone. This is because it functionally integrates the key physiological dilemma of fast-twitch fibers: while they possess a high potential for potentiation, they are also highly fatigable. Consequently, individuals with lower local endurance (indicative of a fast-twitch profile) are expected to accumulate more fatigue, thereby requiring a longer recovery to reveal the PAPE window compared to their high-endurance counterparts.

To test this, the present study employed a heavy back squat as a conditioning stimulus and assessed performance via the Wingate Anaerobic Test (WAnT) for its sensitivity to transient power output (Castañeda-Babarro, 2021). Recovery intervals of 3, 8, and 12 min were selected to probe the critical phases where the balance between fatigue and potentiation shifts. Whereas previous studies have often applied uniform recovery periods, we systematically stratified resistance-trained males based on their 80% 1RM back squat performance to explicitly determine how local muscle endurance capacity influences the optimal recovery time for maximizing PAPE.

We hypothesize that individuals with low local muscle endurance will exhibit a distinct PAPE response, characterized by an optimal recovery window between 8 and 12 min, leading to greater enhancements in peak power output and neuromuscular activity compared to individuals with high local muscle endurance.

## Methods

### Participants

An *a priori* power analysis was performed using G*Power 3.1.9.7 (Faul et al., 2007) to determine the sample size required to detect the interaction effect in a repeated-measures ANOVA (within-between interaction) design. The calculation was conducted for the primary outcome variable, Peak Power (PP). A large effect size (f = 0.40, corresponding to η^2^ = 0.14) was assumed based on similar studies in the PAPE literature (Cohen, 1988), with a correlation of 0.5 among the repeated measurements (the four time points: CON, T3, T8, T12), an alpha (α) level of 0.05, and a statistical power (1 – β) of 0.80. The results indicated that a minimum total sample size of 15 participants was required. To account for potential attrition and ensure robust group comparisons, we recruited 24 participants, exceeding this minimum requirement. Initially, 40 healthy male participants with recreational resistance training experience were recruited from Jiangxi Normal University. The inclusion criteria were: (i) no musculoskeletal injuries within the past 3 months and no diagnosed neuromuscular, cardiovascular, or neurological disorders; (ii) more than 3 years of structured training experience; (iii) at least 2 years of resistance training experience with a back squat 1-repetition maximum (1-RM) greater than 1.5 times body mass; and (iv) engagement in physical activity 2–3 times per week. After a detailed explanation of the experimental procedures and potential risks (Lloria-Varella et al., 2023), 24 eligible participants provided written informed consent and completed all experimental protocols. The study was approved by the Institutional Review Board of Jiangxi Normal University (IRB-JXNU-PEC-2024018) and conducted in accordance with the ethical principles of the Declaration of Helsinki.

The muscle endurance group was determined by an assessment method based on 80% 1RM (Hall et al., 2021; Karp, 2001). Participants were stratified into three groups based on their local muscle endurance performance, as measured by the maximum number of repetitions completed at 80% 1RM in the back squat. According to the criteria established by Karp (2001), individuals completing ≤7 repetitions were classified as the Low-Muscle-Endurance (LME) group, those achieving 8–11 repetitions as the Intermediate-Muscle-Endurance (IME) group, and those completing ≥12 repetitions as the High-Muscle-Endurance (HME) group. The basic information of participants is shown in Table 1.

**TABLE 1:** Participant demographic and anthropometric characteristics.

Group	HME n = 8	IME n = 8	LME n = 8	F	P
Age (years)	20.5 ± 1.2	20.3 ± 1.3	20.1 ± 1.1	0.23	0.80
Height (cm)	176.1 ± 5.2	177.7 ± 3.4	177.3 ± 4.3	0.29	0.75
Body mass (kg)	67.1 ± 7.2	71.1 ± 5.3	70.9 ± 2.8	1.38	0.27
BMI (kg/m^2^)	21.6 ± 2.1	22.5 ± 1.3	22.5 ± 0.5	1.00	0.38
1RM squat (kg)	122.5 ± 28.1	128.2 ± 22.6	136.2 ± 12.2	0.78	0.47
SWR	1.8 ± 0.3	1.8 ± 0.3	1.9 ± 0.2	0.36	0.70
Training experience (years)	5.8 ± 1.5	6.2 ± 1.4	5.5 ± 0.7	0.63	0.54
Completed repetitions at 80% 1RM	14.1 ± 1.1	9.1 ± 1.8	4.9 ± 1.2		

BMI, body mass index; RM, repetition maximum; SWR, relative squat strength.

### Study design

**FIGURE 1:**
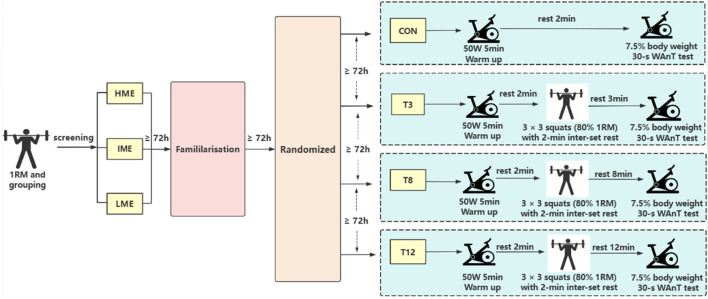
Flowchart of experimental design. Note: RM, repetition maximum; HME, High-Muscle-Endurance; IME, Intermediate-Muscle-Endurance; LME, Low-Muscle-Endurance; CON, baseline; T3, 3 min rest after back squat activation; T8, 8 min rest after back squat activation; T12, 12 min rest after back squat activation; 30 s WAnT, 30-s Wingate Anaerobic test. The same below.

This investigated the effects of different recovery intervals following PAPE (3-min recovery, T3, 8-min recovery, T8, 12-min recovery, T12) on anaerobic performance and neuromuscular activity. Following institutional ethics approval and written informed consent, 24 healthy male participants completed health screenings, anthropometric measurements, standardized exercise technique instruction, and protocol familiarization. The protocol comprised one familiarization session, one control session (CON, which involved no conditioning activity to establish a baseline for comparison), and three PAPE sessions (T3, T8, T12). Each experimental session was separated by a minimum of 72 h to ensure recovery and minimize residual training effects (Figure 1). Sessions were conducted within a consistent 2-h time window relative to each participant’s initial session in an environmentally controlled laboratory (21 °C–24 °C, 44%–56% relative humidity) (Zhang et al., 2024). To minimize confounding variables, participants were instructed to adhere to the following guidelines within the 24 h preceding the experiment: avoidance of high-intensity lower extremity training, abstinence from caffeine and alcohol, and maintenance of habitual nutrition, sleep patterns, and training routines. Additionally, participants were instructed to consume a light, habitual meal at least 3 h before arrival, while strictly avoiding caffeinated beverages, alcohol, and unfamiliar or high-fat foods. They were also required to ensure adequate hydration and rest (Zhang et al., 2024; El-Sayed et al., 2005).

### Procedures

#### One repetition maximum (1RM) test and grouping

Participants first completed a dynamic warm-up using body weight exercises. The 1-RM protocol comprised progressive loading trials (NSCA-National Strength & Conditioning Association, 2021): an initial set of 5–10 repetitions at a submaximal load, followed by 3–5 repetitions at ∼50% of the estimated 1-RM after 1–2 min of rest. Subsequent trials included 2–3 repetitions at 85%–90% of the estimated 1-RM following 2–3 min of recovery. Weight was then increased by 15–20 kg (or 10%–20% of baseline load) for a maximal single attempt after 2–4 min of rest. Load adjustments of 5%–10% were made based on success or failure until the true 1-RM was determined within five attempts. Muscle enduration was estimated through parallel back squats at 80% 1-RM, with participants categorized as (Hall et al., 2021; Karp, 2001): Low-Muscle-Endurance (LME) if completing <7 repetitions, Intermediate-Muscle-Endurance (IME) for 7–12 repetitions, or High-Muscle-Endurance (HME) if exceeding 12 repetitions.

#### Familiarization and warm-up

Participants underwent anthropometric assessments (height, body mass, BMI) and a comprehensive protocol familiarization. This session included individualized cycle ergometer configuration (seat height, handlebar position), practice of the Wingate test protocol to minimize learning effects, and correction of movement techniques (Zhang et al., 2024). Standardized instructions on procedures and safety were provided prior to all experimental sessions. Each trial commenced with an identical warm-up on a calibrated cycle ergometer (CYCLUS 2, Germany), consisting of 5 min of cycling at a self-selected cadence against 50 W resistance. The warm-up incorporated maximal sprints of at least 3 s at the 3-min and 5-min marks, with a target heart rate range of 130–140 beats per minute (Mastalerz et al., 2024; Sauvé et al., 2024).

#### Post-activation performance enhancement (PAPE)

Following the standardized warm-up, participants rested for 10 min off the cycle ergometer. In the PAPE condition, participants performed 3 sets × 3 repetitions of parallel back squats at 80% 1RM with 2-min interset rest, followed by 30-s Wingate anaerobic tests at designated post-intervention timepoints (3, 8, or 12 min). For the CON, participants proceeded directly to the Wingate test after the 10-min rest period without performing squats.

### Surface electromyography selection and placement

Based on the biomechanical characteristics of the squat exercise and the kinematic profiles of the hip, knee, and ankle joints during cycling, supplemented by relevant literature, the primary muscle groups activated were identified. Consequently, the rectus femoris (RF), biceps femoris (BF), and gastrocnemius lateralis (GL) were selected for surface electromyography (sEMG) assessment (Hug and Dorel, 2009). Prior to electrode placement, the skin over the muscle bellies was prepared by shaving to remove hair, lightly abrading with fine-grit sandpaper, and cleansing with 75% alcohol to effectively reduce skin impedance (McManus et al., 2021). In accordance with SENIAM guidelines (Hermens et al., 2000), bipolar disposable Ag/AgCl surface electrodes were placed parallel to the muscle on the designated muscle bellies, with an inter-electrode distance of 2 cm. Electrode placement was verified by having the participant perform brief, low-intensity isometric contractions specific to each muscle. All lead wires were secured with medical adhesive tape to minimize motion artifact. sEMG signals were collected using the Delsys Trigno system. The raw signals were sampled at 1500 Hz, and electrode impedance was confirmed to be below 5 kΩ at the start of data collection. For signal processing, the raw sEMG data were first band-pass filtered between 20 and 450 Hz using a zero-lag fourth order Butterworth filter. Two key parameters were then extracted for analysis: the Integrated Electromyography (IEMG) and the Mean Power Frequency (MPF). The IEMG was calculated by full-wave rectifying the filtered signal followed by numerical integration over the entire cycling trial epoch to represent the total muscle electrical activity. Simultaneously, the MPF was derived by performing a Fast Fourier Transform (FFT) on successive data epochs of the raw signal to obtain the power spectral density, from which the mean frequency was computed.

### Wingate testing

The Wingate test was performed on the same calibrated cycle ergometer (CYCLUS 2, Germany) used for the warm-up. Surface electromyography (sEMG) signals were recorded using a Delsys Trigno system (United States). Following skin preparation (cleansing with alcohol and light abrasion), electrodes were positioned over the RF, BF, and GL (Table 2), and signal stability was verified. The test began with an auditory “go” command, after which participants performed a 30-s maximal sprint against a resistance equivalent to 7.5% of their body mass. Standardized verbal encouragement was provided throughout to ensure maximal effort. The test was conducted according to an established protocol (Liu et al., 2025), starting from a static position with preloaded resistance. Peak power (PP), mean power (MP), and fatigue index (FI) were automatically recorded. Raw sEMG signals were band-pass filtered (10–480 Hz), full-wave rectified, and exported for subsequent analysis.

**TABLE 2:** Muscles studied and EMG electrode placement sites (Hermens et al., 2000; Zipp, 1982).

Muscle	Electrode placement
RF	At 50% of the line from the anterior superior iliac spine to the superior patella
BF	At 50% of the line between the ischial tuberosity and the lateral tibial epicondyle
GL	At approximately 1/3 of the line from the fibular head to the heel

RF, rectus femoris; BF, biceps femoris; GL, gastrocnemius lateralis.

### Index selection

Peak Power (PP) refers to the maximum power output achievable within a brief period, reflecting the capacity for explosive muscular force. It is primarily dependent on the energy supply from the phosphocreatine system, with higher values indicating superior explosive strength. Mean Power (MP) represents the average power maintained throughout the 30-s sprint, serving as an indicator of speed endurance. MP reflects the power output sustained by anaerobic metabolic pathways. Higher mean power values are associated with better speed endurance and greater anaerobic work capacity. The Fatigue Index (FI) quantifies the rate of fatigue development by measuring the decline in power output over time, thereby indicating the body’s ability to resist fatigue under anaerobic conditions (Liu et al., 2025). Integrated electromyography (IEMG) denotes the cumulative electrical activity of motor units over a specific period, which is derived from the amplitude of the EMG signal. It reflects both the number of activated motor units and their firing frequency. Mean Power Frequency (MPF) is defined as the centroid of the power spectrum curve and serves as an effective indicator of muscular activation and functional state. It is also used to assess the degree of muscle fatigue (Shiming et al., 2016; Liu et al., 2025).

### Statistical analysis

Prior to the two-way repeated-measures analysis of variance (ANOVA), the normality of all data distributions was confirmed using Shapiro-Wilk tests. A two-way repeated-measures ANOVA was then employed to examine the effects of muscle endurance group (between-subjects factor: LME, IME, HME), recovery time following PAPE (within-subjects factor: CON, T3, T8, T12), and their interaction on Wingate test-derived anaerobic power indices (PP, MP, FI) and electromyographic (EMG) indices (IEMG, MPF). Where a significant interaction or significant main effect for muscle endurance group was observed (p < 0.05), simple effects analyses were conducted. If only the main effect for muscle endurance group was significant (p < 0.05) and neither the main effect for time nor the interaction was significant (p > 0.05), data were collapsed across muscle endurance groups, and *post hoc* pairwise comparisons with Tukey’s HSD correction for multiple comparisons were performed on the time factor. All statistical analyses were conducted using IBM SPSS Statistics (Version 25.0). Effect sizes were assessed using partial eta-squared ( $\eta_{p}^{2}$ ). Based on conventional criteria, the effects were interpreted as small ( $\eta_{p}^{2}$ = 0.01–0.05), medium ( $\eta_{p}^{2}$ = 0.06–0.13), and large ( $\eta_{p}^{2}$ ≥ 0.14) (Cohen, 1988). Data visualization was performed using GraphPad Prism 10.1, and all raw data were preprocessed using Microsoft Excel 2010. Statistical significance was defined as p < 0.05, with p < 0.01 considered highly significant.

## Results

### Muscle endurance capacity modulated the recovery of anaerobic power

The results of PP, MP and FI after 30 s Wingate test are summarized in Tables 3, 4. Repeated-measures ANOVA of PP during the 30-s Wingate test revealed significant main effects for group (F (2,21) = 5.461, p = 0.012, $\eta_{p}^{2}$ = 0.342) and measurement time (F (3,63) = 5.223, p = 0.003, $\eta_{p}^{2}$ = 0.199), with no significant group × time interaction (F (6,63) = 1.410, p = 0.225, $\eta_{p}^{2}$ = 0.118). Post hoc comparisons confirmed significantly greater PP at T12 than CON (M = 55.80, *SD* = 17.01, 95%CI [6.27,105.32], p = 0.021). Additionally, Tukey’s HSD tests showed a significant difference in PP among groups (F (2, 21) = 15.885, p < 0.001, $\eta_{p}^{2}$ = 0.255). Post hoc comparisons established the following hierarchy: LME produced significantly greater PP than HME (M = 142.87, SD = 25.38, 95% CI [82.43, 203.31], p < 0.001) and IME (M = 77.24, SD = 25.38, 95% CI [16.80, 137.68], p = 0.008). Furthermore, IME resulted in significantly higher PP than ST (M = 65.63, SD = 25.38, 95% CI [5.18, 126.06], p = 0.030).

**TABLE 3:** Wingate test comparison across different muscle endurance and recovery durations.

Wingate test parameters	Group	CON	T3	T8	T12	Main effect of group (average across all times)
		M±SD (CV)	M±SD (CV)	M±SD (CV)	M±SD (CV)	M±SD (CV)
PP(W)	HME	716.2 ± 131.9 (18.4%)	696.4 ± 168.1 (24.2%)	737.6 ± 130.9 (17.7%)	722.3 ± 106.8 (14.8%)	718.1 ± 102.4 (14.3%)
	IEM	728.2 ± 80.4 (11.0%)	781.5 ± 93.5 (12.0%)	823.6 ± 82.8 (10.1%)	801.6 ± 78.0 (9.7%)	783.7 ± 75.6^b^ (9.6%)
	LME	830.2 ± 74.7 (9.0%)	825.9 ± 72.6 (8.8%)	869.6 ± 96.8 (11.1%)	918.0 ± 33.7 (3.7%)	860.9 ± 65.9^b^ ^c^ (7.7%)
	Main effect of time (average across all groups)	758.2 ± 109.1 (14.4%)	767.9 ± 125.1 (ME16.3%)	810.3 ± 114.4 (14.1%)	814.0 ± 112.6^a^ (13.8%)	
MP(W)	HME	593.4 ± 72.3 (12.2%)	568.6 ± 91.2 (16.0%)	593.8 ± 72.8 (12.3%)	584.6 ± 65.6 (11.2%)	585.1 ± 63.6 (10.9%)
	IEM	596.3 ± 61.7 (10.3%)	608.1 ± 40.9 (6.7%)	629.1 ± 61.3 (9.7%)	630.4 ± 59.5 (9.4%)	616.0 ± 49.6 (8.1%)
	LME	603.5 ± 69.6 (11.5%)	629.0 ± 45.1 (7.2%)	637.1 ± 52.7 (8.3%)	639.7 ± 87.9 (13.7%)	627.3 ± 56.3 (9.0%)
	Main effect of time (average across all groups)	597.7 ± 66.2 (11.1%)	601.9 ± 74.6 (12.4%)	620.0 ± 73.6 (11.9%)	618.2 ± 85.4 (13.8%)	
FI(%)	HME	11.9 ± 4.2 (35.3%)	18.0 ± 7.8 (43.3%)	13.1 ± 4.6 (35.1%)	15.3 ± 6.1 (39.9%)	14.6 ± 4.8 (32.9%)
	IEM	16.2 ± 7.7 (47.5%)	16.5 ± 5.3 (32.1%)	17.9 ± 4.3 (24.0%)	16.2 ± 2.6 (16.0%)	16.7 ± 5.5^&^ (32.9%)
	LME	21.2 ± 7.1 (33.5%)	20.0 ± 4.3 (21.5)	20.2 ± 6.5 (32.2%)	20.5 ± 3.8 (18.5%)	20.5 ± 5.3# (25.9%)
	Main effect of time (average across all groups)	16.4 ± 7.3 (44.5%)	18.2 ± 7.1 (39.0%)	17.1 ± 6.1 (35.7%)	17.3 ± 5.9 (34.1%)	

^a^ indicates significant difference compared with CON (all groups).

^b^ indicates significant difference compared with HME (all timepoints).

^c^ indicates significant difference compared with IME (all timepoints).

M±SD, mean ± standard deviation; CV, coefficient of variation; PP, peak power; MP, mean power; FI, fatigue index. The same below.

**TABLE 4:** Analysis results of the effects of group, duration, and their interaction on Wingate Performance.

Condition	Wingate test parameters	df1	df2	F	P	${\;\eta }_{p}^{2}$
Group	PP(W)	2	21	5.461	0.012	0.342
	MP(W)	2	21	1.298	0.294	0.110
	FI(%)	2	21	4.875	0.018	0.377
Duration	PP(W)	3	63	5.223	0.003	0.199
	MP(W)	3	63	1.518	0.229	0.067
	FI(%)	3	63	0.526	0.666	0.024
Group*Duration	PP(W)	6	63	1.410	0.225	0.118
	MP(W)	6	63	0.608	0.672	0.055
	FI(%)	6	63	1.157	0.340	0.099

For MP, no significant main effect of group (F (2, 21) = 1.298, p = 0.294, $\eta_{p}^{2}$ = 0.110), measurement time (F (3,63) = 1.518, p = 0.229, $\eta_{p}^{2}$ = 0.067), or group × time interaction (F (6,63) = 0.608, p = 0.672, $\eta_{p}^{2}$ = 0.055) was observed.

**FIGURE 2:**
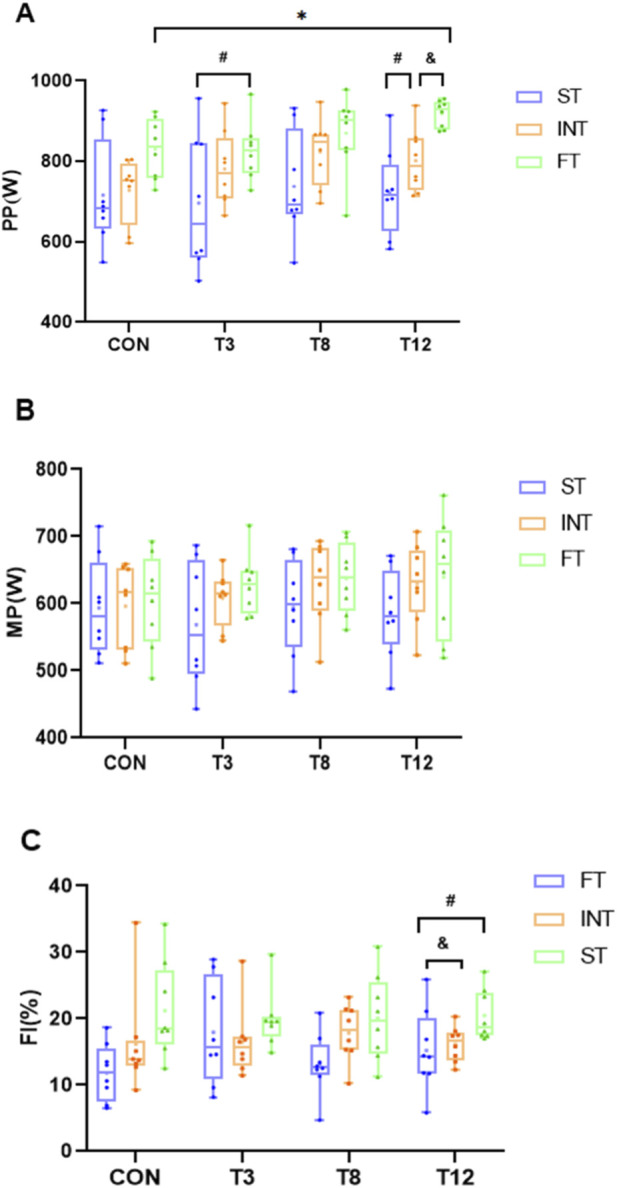
Effects of muscle fiber typology and recovery time on power output and fatigue. Note: * indicates significant difference compared with CON (all groups), # indicates significant difference compared with HME (all times), and indicates significant difference compared with IME (all times). **(A)** Peak power (W), **(B)** Mean power (W), **(C)** Fatigue index (%). The same below.

For FI, repeated-measures ANOVA revealed a significant main effect of group (F (2,21) = 4.875, p = 0.018, $\eta_{p}^{2}$ = 0.377), but no significant main effect of time (F (3,63) = 0.526, p = 0.666, $\eta_{p}^{2}$ = 0.024) or group × time interaction (F (6,63) = 1.157, p = 0.340, $\eta_{p}^{2}$ = 0.099). Given the significant main effect of group in the absence of a significant interaction, *post hoc* analyses were performed on the group main effect. Tukey’s HSD tests indicated that the LME group had a significantly higher fatigue index than both the ST group (M = 5.90, 95% CI [2.60, 9.20], p < 0.001) and the IME group (M = 3.77, 95% CI [0.46, 7.06], p = 0.021) (Figure 2).

## Surface EMG recovery is modulated by muscle endurance capacity

**FIGURE 3:**
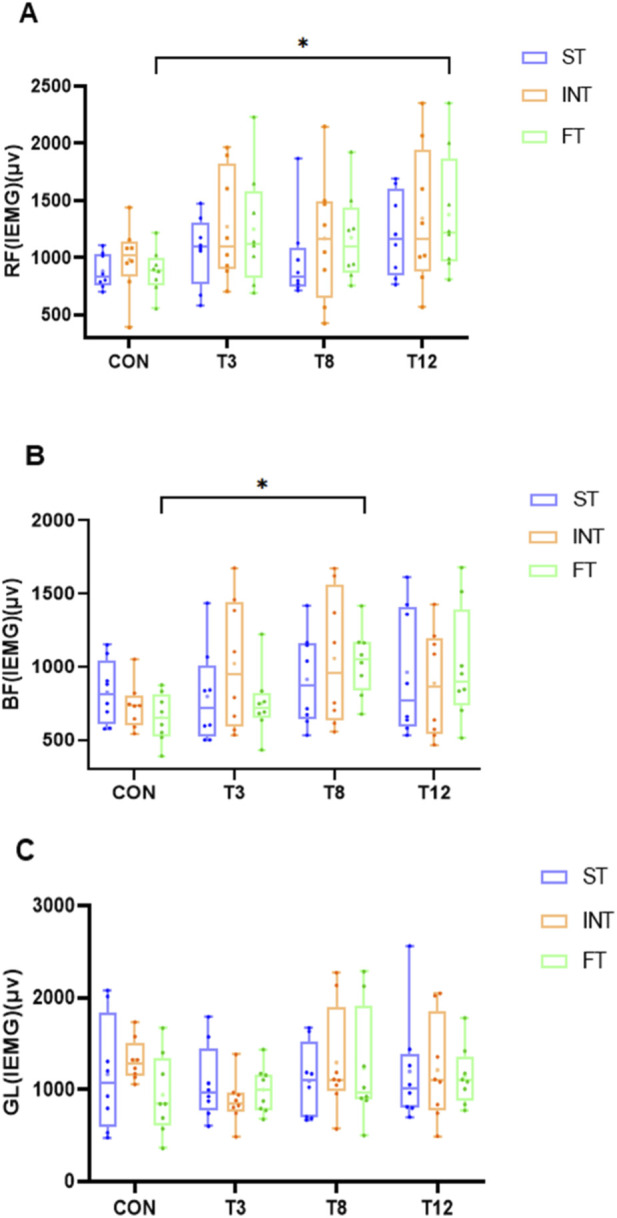
Test results of IEMG with different muscle endurance and recovery durations. **(A)** Rectus femoris IEMG (μV), **(B)** Biceps femoris IEMG (μV), **(C)** Gastrocnemius lateralis IEMG (μV). The same below.

A repeated-measures ANOVA was performed on the IEMG data (Tables 5, 6). For the RF, there were no significant main effects for group (F (2, 21) = 1.186, p = 0.325, $\eta_{p}^{2}$ = 0.102) and group × time interaction (F (6, 63) = 0.109, p = 0.995, $\eta_{p}^{2}$ = 0.010), a significant main effect of time was found (F (3, 63) = 3.790, p = 0.015, $\eta_{p}^{2}$ = 0.153). Post hoc analysis revealed that the IEMG at T12 was significantly higher than that at CON (M = 389.18, SD = 115.98, 95% CI [40.27, 738.10], p = 0.024). For the BF, a significant main effect of time was observed (F (3, 63) = 4.311, p = 0.008, $\eta_{p}^{2}$ = 0.170). Post hoc comparisons indicated that the IEMG at T8 was significantly greater than that at CON (M = 269.67, SD = 75.25, 95% CI [49.40, 489.94], p = 0.011). However, neither the main effect of group (F (2, 21) = 0.168, p = 0.847, $\eta_{p}^{2}$ = 0.016) nor the group × time interaction (F (6, 63) = 1.203, p = 0.317, $\eta_{p}^{2}$ = 0.103) reached statistical significance. For the GL, no significant effects were found for group (F (2, 21) = 0.183, p = 0.834, $\eta_{p}^{2}$ = 0.017), time (F (3, 63) = 1.548, p = 0.221, $\eta_{p}^{2}$ = 0.069), or group × time interaction (F (6, 63) = 0.800, p = 0.574, $\eta_{p}^{2}$ = 0.071) (Figure 3).

**TABLE 5:** Comparison of IEMG from the Wingate test under different muscle endurance and recovery conditions.

Muscle	Group	CON	T3	T8	T12	Main effect of group (average across all times)
		M±SD (CV)	M±SD (CV)	M±SD (CV)	M±SD (CV)	M±SD (CV)
RF	HME	885.3 ± 94.5 (10.7%)	1063.4 ± 184.4 (17.3%)	983.8 ± 190.0 (19.3%)	1199.9 ± 145.5 (12.1%)	1033.1 ± 282.5 (27.3%)
	IEM	986.2 ± 340.9 (34.6%)	1273.9 ± 531.7 (41.7%)	1165.8 ± 205.2 (17.6%)	1342.7 ± 395.2 (29.4%)	1196.4 ± 441.8 (36.9%)
	LME	886.2 ± 228.1 (25.7%)	1247.5 ± 590.1 (38.2%)	1176.9 ± 415.1 (35.3%)	1376.0 ± 345.9 (25.1%)	1174.2 ± 377.0 (28.7%)
	Main effect of time (average across all groups)	919.2 ± 242.1 (26.3%)	1194.9 ± 470.7 (39.4%)	1108.8 ± 289.0 (26.1%)	1306.2 ± 314.7^a^ (24.1%)	
BF	HME	829.8 ± 216.6 (26.1%)	799.3 ± 324.1 (40.5%)	916.0 ± 318.9 (34.8%)	963.1 ± 434.8 (45.1%)	877.1 ± 286.8 (32.7%)
	IEM	734.5 ± 157.3 (21.4%)	1023.9 ± 443.5 (43.3%)	1058.0 ± 458.0 (43.3%)	887.9 ± 371.5 (41.8%)	926.1 ± 328.4 (35.5%)
	LME	654.2 ± 152.9 (27.1%)	753.4 ± 242.4 (32.2%)	1039.1 ± 245.9 (23.7%)	1007.6 ± 428.2 (42.5%)	860.0 ± 237.2 (27.6%)
	Main effect of time (average across all groups)	739.5 ± 178.0 (24.1%)	858.9 ± 392.7 (45.7%)	1004.4 ± 352.1^a^ (35.1%)	952.9 ± 413.3 (43.4%)	
GL	HME	1165.3 ± 591.3 (50.7%)	1072.4 ± 441.2 (41.1%)	1096.7 ± 500.3 (45.6%)	1198.9 ± 579.4 (48.3%)	1133.3 ± 501.3 (44.2%)
	IEM	1318.4 ± 164.6 (12.5%)	878.9 ± 252.3 (28.7%)	1297.8 ± 475.1 (36.6%)	1214.0 ± 350.3 (28.9%)	1177.3 ± 310.6 (26.4%)
	LME	943.3 ± 474.9 (50.3%)	998.6 ± 388.4 (38.9%)	1234.5 ± 583.0 (47.2%)	1148.9 ± 350.3 (30.4%)	1081.3 ± 425.9 (39.4%)
	Main effect of time (average across all groups)	1142.3 ± 448.1 (39.2%)	982.1 ± 387.4 (39.4%)	1209.7 ± 521.3 (43.1%)	1187.3 ± 430.4 (36.3%)	

^a^ indicates significant difference compared with CON (all groups). RF, rectus femoris; BF, biceps femoris; GL, gastrocnemius lateralis.

**TABLE 6:** Analysis results of the effects of group, duration, and their interaction on IEMG.

Condition	Muscle	df1	df2	F	P	${\;\eta }_{p}^{2}$
Group	RF	2	21	1.186	0.325	0.102
	BF	2	21	0.168	0.847	0.016
	GL	2	21	0.183	0.834	0.017
Duration	RF	3	63	3.790	0.015	0.153
	BF	3	63	4.311	0.008	0.170
	GL	3	63	1.548	0.221	0.069
Group*Duration	RF	6	63	0.109	0.995	0.010
	BF	6	63	1.203	0.317	0.103
	GL	6	63	0.800	0.574	0.071

**FIGURE 4:**
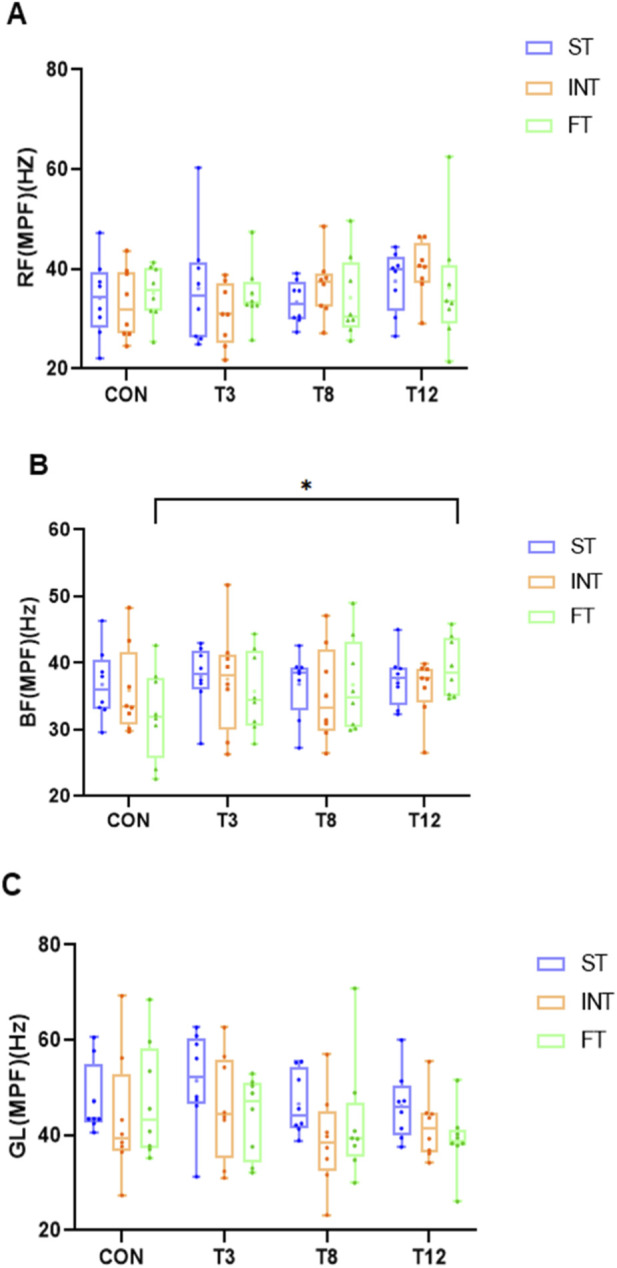
Test results of MPF with different muscle endurance and recovery durations. **(A)** Rectus femoris MPF (Hz), **(B)** Biceps femoris MPF (Hz), **(C)** Gastrocnemius lateralis MPF (Hz). The same below.

As shown in Tables 7, 8, a repeated-measures ANOVA on MPF revealed muscle-specific patterns. Non-significant group main effects were observed for RF (*F* (2,21) = 0.003, p = 0.997, $\eta_{p}^{2}$ < 0.001), BF (*F* (2,21) = 0.076, p = 0.927, $\eta_{p}^{2}$ = 0.007), and GL (*F* (2,21) = 2.561, p = 0.101, $\eta_{p}^{2}$ = 0.196). While time effects were non-significant for RF (*F* (3,63) = 1.568, p = 0.206, $\eta_{p}^{2}$ = 0.069) and GL (*F* (3,63) = 1.812, p = 0.154, $\eta_{p}^{2}$ = 0.079), BF exhibited a significant time main effect (*F* (3,63) = 2.930, p = 0.040, $\eta_{p}^{2}$ = 0.122). No significant group × time interactions emerged for any muscle (p > 0.05). Post hoc comparisons confirmed significantly greater MPF at T12 than CON (M = 3.23, *SD* = 1.07, 95% CI [ 0.12, 6.35], p = 0.039) (Figure 4).

**TABLE 7:** Comparison of MPF from the Wingate test under different muscle endurance and recovery conditions.

Muscle	Group	CON	T3	T8	T12	Main effect of group (average across all times)
		M±SD (CV)	M±SD (CV)	M±SD (CV)	M±SD (CV)	M±SD (CV)
RF	HME	34.2 ± 7.8 (22.8%)	36.1 ± 11.7 (32.4%)	33.3 ± 4.3 (12.9%)	42.4 ± 6.9 (5.48%)	35.3 ± 6.4 (18.1%)
	IEM	33.1 ± 7.0 (21.1%)	30.8 ± 6.2 (20.1%)	36.6 ± 6.3 (17.2%)	40.0 ± 5.5 (13.4%)	35.1 ± 5.8 (16.5%)
	LME	35.2 ± 5.5 (15.6%)	34.8 ± 6.1 (17.5%)	34.2 ± 6.8 (19.9%)	36.2 ± 12.2 (33.7%)	35.1 ± 6.4 (18.2%)
	Main effect of time (average across all groups)	34.2 ± 6.6 (19.3%)	33.9 ± 8.5 (25.1%)	34.7 ± 5.7 (16.4%)	37.9 ± 8.2 (21.6%)	
BF	HME	36.8 ± 5.3 (14.4%)	39.2 ± 2.6 (6.63%)	38.1 ± 3.2 (8.4%)	37.8 ± 2.8 (7.4%)	38.0 ± 3.2 (8.4%)
	IEM	35.9 ± 6.5 (18.1%)	39.3 ± 4.6 (11.7%)	36.5 ± 6.1 (16.7%)	38.5 ± 1.7 (4.4%)	37.6 ± 4.6 (12.2%)
	LME	33.3 ± 4.5 (13.5%)	37.0 ± 5.0 (13.5%)	38.0 ± 6.5 (17.1%)	40.4 ± 2.8 (6.9%)	37.2 ± 4.9 (10.8%)
	Main effect of time (average across all groups)	35.3 ± 5.6 (14.2%)	38.5 ± 4.0 (10.4%)	37.5 ± 5.2 (13.9%)	38.9 ± 2.6* (5.1%)	
GL	HME	47.3 ± 7.4 (15.6%)	51.5 ± 10.3 (20.0%)	46.6 ± 6.5 (13.9%)	46.1 ± 7.2 (15.6%)	47.9 ± 7.5 (15.7%)
	IEM	43.6 ± 7.8 (29.8%)	46.1 ± 11.2 (24.3%)	38.9 ± 10.0 (25.7%)	41.8 ± 6.8 (16.3%)	42.6 ± 10.0 (23.5%)
	LME	47.3 ± 12.0 (25.4%)	43.9 ± 8.4 (19.1%)	41.5 ± 9.5 (22.9%)	39.2 ± 6.9 (17.6%)	43.0 ± 8.9 (20.7%)
	Main effect of time (average across all groups)	46.1 ± 10.7 (23.2%)	47.2 ± 10.0 (21.2%)	42.3 ± 8.6 (20.3%)	42.4 ± 6.9 (16.3%)	

**TABLE 8:** Analysis results of the effects of group, duration, and their interaction on MPF.

Condition	Muscle	df1	df2	F	P	${\;\eta }_{p}^{2}$
Group	RF	2	21	0.003	0.997	<0.001
	BF	2	21	0.076	0.927	0.007
	GL	2	21	2.561	0.101	0.196
Duration	RF	3	63	1.568	0.206	0.069
	BF	3	63	2.930	0.040	0.122
	GL	3	63	1.812	0.154	0.079
Group*Duration	RF	6	63	0.763	0.602	0.068
	BF	6	63	0.862	0.528	0.076
	GL	6	63	0.405	0.873	0.037

## Discussion

This study systematically investigated neuromuscular responses to PAPE in resistance-trained males grouped based on muscle endurance. The 30-s Wingate Anaerobic Test (WAnT) was selected over shorter, high-intensity tests (e.g., vertical jumps or short sprints) for a key methodological reason: it allows for the simultaneous evaluation of both peak power output (a key marker of PAPE) and the subsequent fatigue profile within a single, standardized maximal effort. While shorter tests are excellent isolated measures of explosive power, the WAnT uniquely captures the dynamic interplay between potentiation and fatigue, a central aspect of PAPE physiology (Doma et al., 2019; Peñailillo et al., 2013; Ericson et al., 1986). This was particularly relevant for our study, as we hypothesized that individuals with a fast-twitch tendency (inferred via low local muscle endurance) would not only exhibit greater potentiation but also a distinct fatigue profile. The WAnT provided the necessary data granularity (Peak Power, Mean Power, and Fatigue Index) to test this hypothesis comprehensively, offering insights into the time-course of performance changes that a single, brief explosive test could not. Furthermore, in terms of reproducibility, which is crucial in repeated-measures designs such as ours, the WAnT has been widely established as a tool with high test-retest reliability for peak power in trained populations. Although a formal reliability analysis was not a primary aim of our study, its consistency is well documented in the literature (Zupan et al., 2009; Mendez-Villanueva et al., 2007; Paton and Hopkins, 2001). We have added a sentence in the Methods section to acknowledge this established reliability. A central finding was the critical influence of muscle endurance capacity on PAPE. This was demonstrated by a clear, hierarchical enhancement in peak power output across groups (LME > IME > HME). The large effect sizes (≥0.34) for these between-group differences in PP and FI underscore that an individual’s muscle endurance profile is a substantial physiological determinant of both the magnitude of PAPE and the rate of fatigue. This finding extends beyond statistical significance, highlighting its practical relevance for personalizing training regimens. LME group generated significantly greater power than HME and IEM groups, attributable to enhanced glycolytic capacity, accelerated sarcoplasmic reticulum Ca^2+^ release kinetics, and elevated myosin ATPase activity (Sale, 2002; Bottinelli et al., 1996). Paradoxically, this power advantage was accompanied by significantly elevated fatigue index (FI) in LME versus HME and IME groups, as a direct consequence of accelerated metabolite accumulation and sustained energy demands from myosin light chain phosphorylation. Notably, no significant between-group differences occurred in MP during the 30-s Wingate test (p > 0.05). This equivalence stems from compensatory mechanisms: the initial power advantage in LME individuals was offset by accelerated fatigue, while HME participants maintained output via superior oxidative capacity and lactate clearance (Schiaffino and Reggiani, 2011; Brooks, 2018). Moreover, differences in phosphocreatine (PCr) resynthesis rates and lactate shuttle dynamics, mediated by fiber-specific expression of monocarboxylate transporters (e.g., MCT4 in type II fibers), further modulated the net metabolic response and fatigue resistance between groups. Consequently, convergent neuromuscular strategies yielded comparable time-integrated power across groups during sustained anaerobic efforts. Methodologically, the 30-s WAnT test duration represents a critical transition phase (15–45 s) where neither purely anaerobic (<15 s) nor aerobic-dominant (>45 s) energy systems prevail, attenuating inherent fiber-type advantages (Gastin, 2001; Harris et al., 1976). HME participants leveraged inherent oxidative advantages, which facilitate rapid lactate shuttling as a metabolic fuel and signal, and drive the high-rate resynthesis of phosphocreatine (PCr) (Glancy et al., 2021). In contrast, LME exhibited comparable recovery rates, potentially due to training-induced adaptations such as enhanced mitochondrial biogenesis within type II fibers and increased expression of monocarboxylate transporter 4 (MCT4), which facilitates lactate efflux (Bonen, 2000). These metabolic adaptations, along with variations in central drive and motor unit recruitment thresholds, may partially compensate for the inherently lower oxidative capacity of LME, reducing inter-group differences in recovery kinetics during the WAnT.

Electromyographic analysis indicated a biphasic PAPE response in the RF and BF, albeit with distinct temporal profiles. For the BF, IEMG amplitude increased significantly at T8 compared with the CON condition. In contrast, the RF exhibited a significant IEMG elevation later, at T12 relative to CON. This was accompanied by an increase in MPF at T12, suggesting improved motor unit firing synchrony. The differential timing of PAPE emergence between the RF and BF corresponds to their functional roles during the squat: the BF serves as a primary hip extensor and knee stabilizer, activating earlier and more intensely, while the RF, as a bi-articular muscle involved in both hip flexion and knee extension, may demonstrate delayed potentiation (Neumann, 2010). No significant PAPE effects were observed in the GL, likely due to its submaximal recruitment during the back squat (Escamilla et al., 1998; Bryanton et al., 2012). Additionally, the GL typically exhibits a higher oxidative capacity and a greater proportion of type I fibers compared to more glycolytic muscles like the rectus femoris (RF), as supported by established data on fiber-type distribution and metabolic enzyme activity in human lower limb muscles (Johnson et al., 1973; Ariano et al., 1973). This inherent characteristic may reduce its susceptibility to the rapid potentiation mechanisms that are more pronounced in muscles with a higher glycolytic potential. The biphasic IEMG–MPF response observed in the RF and BF aligns with phosphorylation-dependent mechanisms that are predominantly active in type II fibers. The initial increase in IEMG may indicate enhanced excitation–contraction coupling mediated by rapid Ca^2+^ release (Schiaffino and Reggiani, 2011). In contrast, the subsequent rise in MPF could be attributed to phosphorylation of the myosin regulatory light chain, which improves cross-bridge cycling kinetics (Rose and Hargreaves, 2010). The pronounced fatigability of type II fibers after intense exercise may be related to their high metabolic demand and relatively inefficient calcium reuptake (Allen et al., 2008). Although MPF is influenced by muscle fiber conduction velocity and can be altered by fatigue, its utility in discriminating fiber-type composition remains limited. This is due to spatial averaging over heterogeneous muscle regions and confounding factors such as motor unit synchronization (Piperi et al., 2024; Brad and Sherry, 2004). No significant among-group differences in recovery kinetics were observed (p > 0.05). The absence of PAPE at T3 is consistent with literature indicating that 8 min of recovery are generally needed to overcome metabolic fatigue and allow potentiation to dominate (Garbisu-Hualde and Santos-Concejero, 2021). The high-intensity squat protocol (3 × 3 repetitions at 80% 1RM) likely induced substantial fatigue, masking any early potentiation at shorter recovery intervals (Jo et al., 2010; Chen et al., 2023). Individual differences in central drive, motor unit recruitment strategy, and metabolic recovery (e.g., PCr resynthesis, lactate clearance) further modulated the fatigue–potentiation balance, likely explaining the lack of inter-group differences.

A notable aspect of this study is the implementation of a practical, performance-based stratification framework, employing repetition capacity at 80% 1RM in the back squat as a surrogate indicator of fiber-type dominance as a proxy for fiber-type dominance to guide PAPE timing. Our findings reveal that individuals classified as fast-twitch-dominant reached peak performance enhancement at approximately 12 min, while those identified as slow-twitch-dominant exhibited markedly attenuated responses. These results support extending the recovery window to 8–12 min for fast-twitch-dominant individuals to optimize PAPE, thereby refining current guidelines that often overlook interindividual differences in muscle typology. It must be acknowledged, however, that this repetition-based approach offers only an approximate estimation of fiber-type distribution. It cannot distinguish between subtype variations (e.g., type IIa vs. IIx) or confirm actual physiological composition in the absence of direct biological validation such as muscle biopsy, magnetic resonance spectroscopy, or advanced imaging. Nevertheless, the clear response differences observed between stratification groups underscore the utility of this method in tailoring recovery strategies. Substantial inter-individual heterogeneity was observed within the study cohort, particularly with respect to sEMG-derived parameters, for which coefficients of variation frequently exceeded 50%. The persistence of such variability even after stratification by the primary physiological determinant of muscle endurance suggests the involvement of additional modulating factors. These are likely to encompass inter-individual differences in training adaptations, neuromotor coordination, central activation, and the kinetics of key metabolic processes such as phosphocreatine resynthesis and lactate clearance. Therefore, although fiber typology offers a robust framework for predicting group-level responses, our findings ultimately underscore the necessity of individualized PAPE protocols, with recovery strategies tailored according to specific athlete characteristics and physiological responses.

## Conclusion

This study demonstrates that a heavy back squat conditioning activity elicits a significant PAPE effect, with the optimal window for enhanced anaerobic performance and neuromuscular activity occurring between 8 and 12 min of recovery. The findings reveal a muscle-specific pattern of potentiation, with the biceps femoris demonstrating earlier (T8) and the rectus femoris later (T12) elevations in neuromuscular activity. Crucially, muscle endurance capacity, as stratified by performance at 80% 1RM, was identified as a pivotal moderator of the PAPE response. Individuals with low muscle endurance (LME) exhibited superior peak power output and a more pronounced PAPE effect compared to their high-endurance (HME) counterparts, albeit with a concomitant higher fatigue index. This establishes muscle endurance profile as a key determinant for individualizing recovery strategies to optimize post-activation performance enhancement.

## Limitations

Despite the insightful findings, this study has several limitations. First, the classification of muscle fiber dominance was based on the maximum repetitions at 80% 1RM. While this is a validated and practical non-invasive method, it remains an indirect proxy and can be influenced by factors such as neuromuscular efficiency and technique, unlike direct histological analysis. Second, although participants were instructed to maintain their habitual diet and avoid specific substances, the precise nutritional intake and hydration status were not monitored or controlled, which could have introduced variability in metabolic and performance responses. Third, the relatively small and homogeneous sample of recreationally trained young males limits the generalizability of our findings to other populations, including females, elite athletes, and older adults. Future research should incorporate more direct fiber-type assessments, include more diverse cohorts, and implement stricter nutritional control to further elucidate the individualized nature of PAPE kinetics.

## Data Availability

The raw data supporting the conclusions of this article will be made available by the authors, without undue reservation.
